# Does spacer type influence bone lysis and clinical outcomes? A comparative analysis in two-stage revision for periprosthetic knee infection

**DOI:** 10.1186/s12893-026-03883-3

**Published:** 2026-05-29

**Authors:** Cihan Ünyılmaz, Cem Çopuroğlu, Mert Özcan, Ozan Güner

**Affiliations:** Department of Orthopaedics and Traumatology, Trakya University Faculty of Medicine, Edirne, Türkiye

**Keywords:** Periprosthetic joint infection, Knee arthroplasty, Spacer, Bone lysis, AORI score, Two-stage revision, Handmade spacer, Prefabricated spacer, Radiological outcome, Functional outcome

## Abstract

**Background:**

Periprosthetic joint infection (PJI) following total knee arthroplasty remains a serious complication associated with substantial morbidity and complex surgical management. Two-stage revision arthroplasty is widely accepted as the gold standard treatment, in which an antibiotic-loaded cement spacer is used during the interim period to maintain joint space and deliver local antimicrobial therapy. While previous studies have compared static and articulating spacers, limited data are available regarding the impact of handmade versus prefabricated spacers on radiological bone loss and functional outcomes.

**Methods:**

This retrospective observational study included 23 patients who underwent two-stage revision surgery for knee PJI between 2011 and 2024. PJI was diagnosed according to the 2018 International Consensus Meeting (ICM) criteria. Patients were divided into two groups based on spacer type: handmade static vancomycin-loaded spacers (*n* = 14) and prefabricated articulating gentamicin-loaded spacers (*n* = 9). Clinical outcomes (reinfection, complications), functional outcomes (range of motion [ROM], WOMAC score, ambulation status), and radiological parameters (bone lysis, joint space gap measurements, and AORI classification) were compared between groups. Statistical analyses were performed using nonparametric tests, with significance set at *p* < 0.05.

**Results:**

Reinfection rates were higher in the handmade spacer group (28.6%) compared with the prefabricated group (11.1%), although the difference was not statistically significant. Radiological analysis suggested greater femoral and tibial bone lysis in the handmade spacer group, reflected by significantly higher AORI scores (femoral *p* = 0.005; tibial *p* = 0.002). Functional outcomes favored the prefabricated spacer group, with significantly improved postoperative ROM (*p* = 0.007) and WOMAC scores (*p* = 0.038). Independent ambulation was achieved more frequently in the prefabricated spacer group (77.8% vs. 21.4%, *p* = 0.019). Complication rates, including spacer failure and salvage procedures, were also higher in the handmade spacer group.

**Conclusion:**

In this retrospective cohort, the clinical pathway involving prefabricated articulating gentamicin-loaded spacers was associated with less radiological bone loss and improved functional outcomes compared with handmade static vancomycin-loaded spacers during two-stage revision for knee PJI. However, given the limited sample size and retrospective design, these findings should be interpreted cautiously and require confirmation in larger prospective studies.

Periprosthetic joint infection (PJI) of the knee represents one of the most challenging complications following total knee arthroplasty (TKA), frequently leading to significant morbidity, the need for repeated surgical interventions, and, in some cases, increased mortality. This condition results from microbial colonization and subsequent biofilm formation on prosthetic components, which reduces the efficacy of conventional antibiotic therapies and complicates infection management. The reported incidence of PJI after primary TKA ranges from 1 to 2%, posing a considerable clinical and economic burden on healthcare systems worldwide [1–3].

The management of PJI is complex and requires a multidisciplinary approach. Among the available treatment options, two-stage revision arthroplasty remains the gold standard, particularly for chronic or refractory infections.

The first stage involves removal of the infected prosthesis and cement, extensive debridement of infected tissues, and implantation of an antibiotic-impregnated cement spacer. This is followed by intravenous antibiotic therapy aimed at eradicating the infection. Once infection control is confirmed both clinically and microbiologically, the second stage entails spacer removal, further debridement, and implantation of a new prosthesis. Infection eradication rates for two-stage revisions have been reported between 85% and 96%, with favorable long-term outcomes when combined with appropriate antimicrobial therapy and meticulous surgical planning [4–7].

During the interim period between stages, spacers serve two critical functions: they deliver high local concentrations of antibiotics and maintain joint space, soft tissue tension, and limb alignment, thus facilitating reimplantation.

Depending on patient condition and bone loss severity, spacers may be static or articulating. Static spacers are generally preferred in cases with extensive bone defects or poor soft tissue support, while articulating spacers allow limited joint motion and promote better postoperative functional recovery. Although infection eradication rates are similar between spacer types, articulating or prefabricated spacers have been consistently associated with improved range of motion and higher patient satisfaction [8, 9].

Although many studies compare static versus articulating spacers, fewer data quantify radiological bone resorption specifically between handmade and prefabricated spacer constructs. The association between spacer design and bone lysis during the interim period remains incompletely characterized. Therefore, we evaluated clinical, functional, and radiological outcomes, focusing on radiographic bone lysis quantified with standardized measurements and reported using AORI classification.

Despite their essential role in infection management, the long-term radiological and structural effects of different spacer types on bone integrity remain insufficiently clarified.

Therefore, this study aims to compare the clinical, functional, and radiological outcomes of handmade and prefabricated spacers used in two-stage revision surgeries for periprosthetic knee infections.

Particular emphasis was placed on infection eradication rates, complication profiles, and the degree of bone resorption (lysis), in order to evaluate the influence of spacer type on surgical planning and long-term joint preservation. We hypothesized that prefabricated spacers would be associated with less radiographic bone lysis and better interim functional outcomes compared with handmade spacers.

This study aimed to address the following research questions:

1. Does the type of spacer (handmade vs. prefabricated) affect infection eradication and reinfection rates?
2. Does spacer type influence the degree of bone resorption (lysis) and AORI classification scores?
3. Are there differences in functional outcomes, including range of motion (ROM), WOMAC scores, and ambulation status, between spacer types?
4. Does spacer type impact the incidence and severity of postoperative complications?

## Materials and methods

### Study design and ethical approval

This retrospective observational study was conducted at the Department of Orthopaedics and Traumatology, Trakya University Faculty of Medicine.

This study was approved by the Trakya University Faculty of Medicine Non-Interventional Clinical Research Ethics Committee (Approval No: 2025/164; Date: September 8, 2025).

All procedures adhered to the Declaration of Helsinki, and all patient data were anonymized prior to analysis.

### Patient selection and grouping

Between 2011 and 2024, 25 patients who underwent two-stage revision surgery for periprosthetic knee infection were reviewed.

Two patients were excluded — one due to loss to follow-up after spacer implantation and another due to death during the spacer period. PJI was diagnosed according to the 2018 ICM criteria. Cases were classified as acute or chronic PJI based on symptom duration and timing from index arthroplasty.

The final study cohort included 23 patients with complete clinical and radiological data and at least one year of follow-up. Patients with sinus tract or significant soft tissue defects were excluded to reduce baseline heterogeneity.

Patients were divided into two groups according to the type of spacer used:

- Handmade Spacer Group (n=14)
- Prefabricated Spacer Group (n=9)

### Spacer preparation and surgical procedure

In all cases, the first-stage surgery involved removal of the infected prosthesis and cement, extensive debridement, and insertion of an antibiotic-loaded cement spacer.

Handmade spacers were molded intraoperatively using polymethylmethacrylate (PMMA) bone cement loaded with vancomycin (2 g per 40 g cement), manually shaped around an intramedullary rod. The handmade spacer was constructed as a static spacer using an intramedullary rod for structural support.

Prefabricated spacers were commercially available articulating devices (Spacer-K^®^, Tecres S.p.A., Italy) containing gentamicin-loaded PMMA, chosen according to knee size. Handmade spacers contained vancomycin-loaded PMMA, whereas prefabricated spacers contained gentamicin-loaded PMMA; this difference in antibiotic composition represents a potential confounder.

After spacer implantation, targeted intravenous antibiotic therapy was administered for at least six weeks, based on intraoperative culture results and infectious disease consultation.

Once infection eradication was confirmed both clinically and via laboratory markers, the second-stage revision total knee arthroplasty was performed.

*At the second-stage reimplantation*,* the same revision knee prosthesis design with the same level of constraint was used in all patients according to the institutional surgical protocol. Therefore*,* implant type did not represent a confounding factor for postoperative functional outcome comparisons.* Spacer type was selected based on surgeon preference and implant availability; predefined selection criteria were not applied. Spacer type was selected according to surgeon preference and implant availability rather than predefined clinical criteria. No standardized allocation protocol was used to assign patients based on case complexity or infection severity. Reimplantation was performed when clinical signs of infection had resolved and inflammatory markers (CRP/ESR) had normalized or showed a sustained downward trend, in conjunction with infectious disease consultation.

### Data collection

Clinical, radiological, and laboratory data were retrospectively collected from hospital records, radiology archives, and outpatient follow-ups. Comorbidities (e.g., diabetes, chronic renal disease) and immunosuppressive status were recorded when available. Reinfection was defined according to the 2018 International Consensus Meeting (ICM) criteria, with a score of ≥ 6 indicating infection. In addition, clinical findings, laboratory parameters, and the need for further surgical intervention were also considered in accordance with established clinical guidelines. Spacer-period failure was defined as any condition requiring unplanned surgical intervention during the interval period, including persistent infection or mechanical complications. Salvage procedures were defined as interventions performed in cases of persistent infection, severe bone loss, or failure of the standard two-stage revision strategy.

#### Demographic parameters

- Age, sex, body mass index (BMI)
- Spacer retention time (days) and total follow-up duration (months)

#### Laboratory parameters

- C-reactive protein (CRP, mg/L)
- Erythrocyte sedimentation rate (ESR, mm/hour)
- White blood cell count (WBC, /µL)

All were assessed before spacer placement and prior to reimplantation.

#### Functional evaluation

- Range of motion (ROM): recorded after spacer insertion and after reimplantation
- WOMAC score: assessing pain, stiffness, and physical function
- Ambulation level: categorized as independent, assisted, or immobile

#### Microbiology

- Intraoperative cultures were recorded, including organism type and available antibiotic susceptibility profiles.

### Radiological evaluation

Radiographic measurements were performed using calibrated anteroposterior (AP) knee radiographs obtained immediately after spacer placement and just before reimplantation.

All measurements were performed by an experienced orthopaedic surgeon using a calibrated digital imaging system with standardized anatomical reference points. In our institution, all radiographs are routinely obtained using standardized anteroposterior knee positioning protocols and consistent imaging distances, which may have improved comparability between early and late radiographs. Due to the retrospective design, the assessor was not formally blinded to group allocation; however, radiographs were evaluated in a mixed and non-sequential manner rather than in chronological order to reduce potential measurement bias.

### Bone lysis (resorption)

**Fig. 1:**
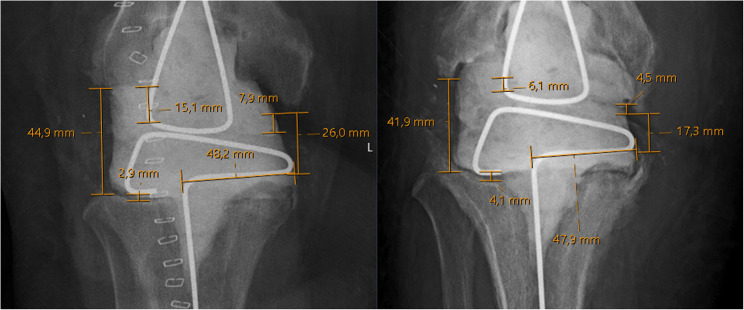
Radiographic assessment of femoral and tibial bone loss using standardized cortical reference points on anteroposterior knee radiographs. Measurements were performed by calculating the distance between defined cortical landmarks of the femur and tibia and the spacer interface. The same anatomical reference points were consistently used at both time points (immediately after spacer placement and prior to reimplantation) to ensure reproducibility and comparability of measurements

**Fig. 2:**
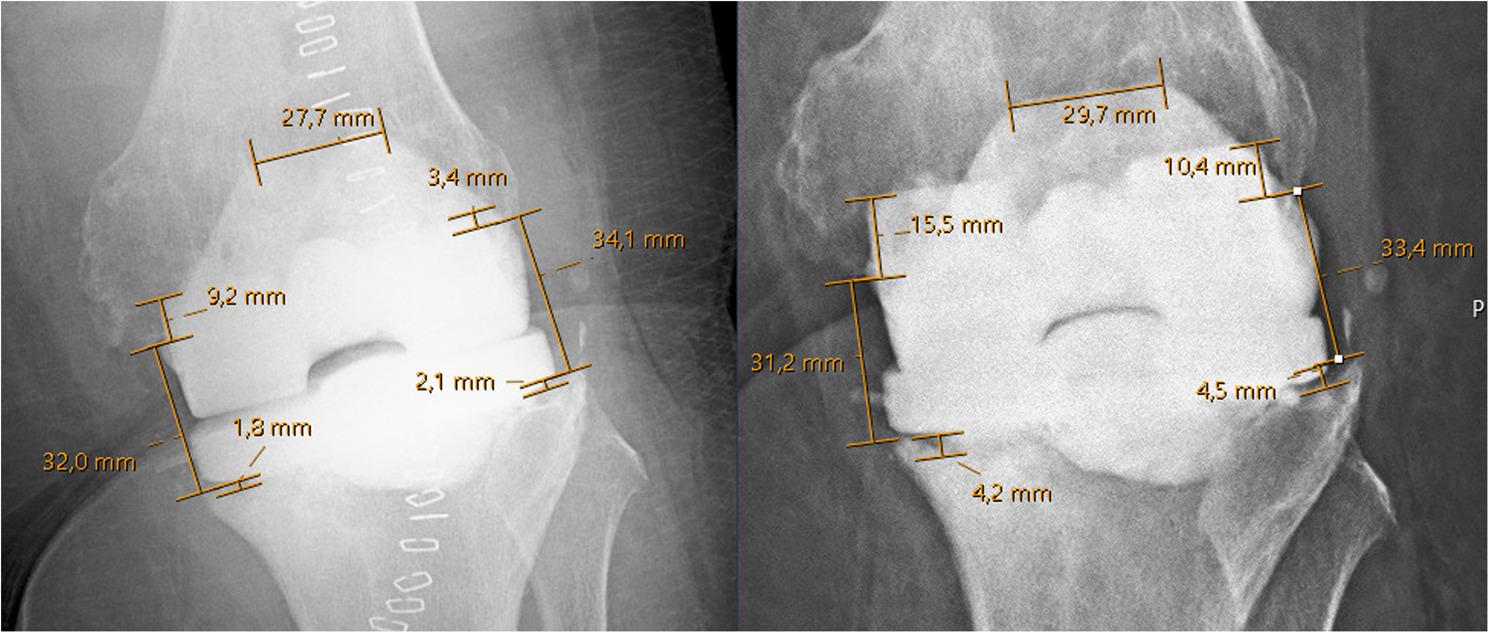
Radiographic assessment of femoral and tibial bone loss using standardized cortical reference points on anteroposterior knee radiographs. Measurements were performed by calculating the distance between defined cortical landmarks of the femur and tibia and the spacer interface. The same anatomical reference points were consistently used at both time points (immediately after spacer placement and prior to reimplantation) to ensure reproducibility and comparability of measurements

The degree of femoral and tibial bone loss was calculated by measuring the distance between cortical reference points on the medial and lateral condyles (femur) and plateaus (tibia) relative to the spacer interface.(Figures 1 and 2).

### Joint space narrowing (gap measurements)

- Medial Gap: measured between fixed reference points on the medial femoral condyle and tibial plateau.
- Lateral Gap: measured between the lateral femoral condyle and the fibular head.Differences between early and late radiographs were calculated to determine joint space preservation.
- Differences between early and late radiographs were calculated to determine joint space preservation.

### AORI bone defect classification

The Anderson Orthopaedic Research Institute (AORI) classification was used to categorize femoral and tibial bone loss separately (F1–F3 and T1–T3) [10]. AORI classification was applied at two time points—immediately after spacer placement and prior to reimplantation—to evaluate changes in bone loss during the interim period.

### Surgical outcomes and complications

All patients underwent second-stage revision total knee arthroplasty after infection control.

Two patients developed long-term complications: one required Ilizarov arthrodesis and another underwent osteobridge reconstruction.

During the spacer period, two patients experienced wound-healing problems, one of whom required flap coverage.

### Data analyses

All statistical analyses were performed using SPSS version 26.0 (IBM Corp., Armonk, NY, USA).

Continuous variables were presented as mean ± standard deviation (SD) and categorical variables as counts and percentages.

*The* *Shapiro–Wilk test* was used to assess normality.

The *Mann–Whitney U test* was applied for non-normally distributed data, and *Chi-square* or *Fisher’s exact tests* for categorical variables.

Paired comparisons between pre- and post-spacer parameters were analyzed with the *Wilcoxon signed-rank test*.

Correlations between AORI scores and functional outcomes (ROM, WOMAC) were evaluated using the *Spearman* rank correlation.

A *P*-value < 0.05 was considered statistically significant.

Given the retrospective design and limited cohort size, a formal a priori power analysis was not performed and the statistical analyses should be interpreted as exploratory.

## Results

A total of 23 patients who underwent two-stage revision surgery for periprosthetic knee joint infection were included in the study. Fourteen patients received handmade spacers, and nine patients received prefabricated spacers. The groups were compared in terms of demographic, laboratory, radiological, and functional parameters. Statistical significance was set at *p* < 0.05. All patients underwent reimplantation using the same definitive revision implant design and constraint level.

### Demographic characteristics

There were no significant differences between the groups regarding age (62.6 vs. 66.1 years, *p* = 0.295) or sex distribution (71.4% vs. 77.8% female, *p* = 0.295).

The mean body mass index (BMI) was significantly higher in the handmade spacer group (33.67 ± 0.6 kg/m²) compared to the prefabricated spacer group (29.14 ± 2.1 kg/m²; *p* = 0.009). This baseline difference should be noted, as increased BMI may act as a potential confounder for postoperative functional outcomes, mechanical loading during the spacer period, and wound-related complications.

Although the spacer retention period was longer in the handmade group (120.7 ± 13.3 days) than in the prefabricated group (91.8 ± 13.4 days), this difference did not reach statistical significance (*p* = 0.071).

No significant differences were found in follow-up duration between the two groups (Table 1).

**Table 1:** .

Parameter	Handmade Spacer (*n* = 14)	Preformed Spacer (*n* = 9)	*p*-value
Age (years)	62.6 ± 5.3	66.1 ± 5.3	0.295
Sex (Female, %)	71.4%	77.8%	0.295
BMI (kg/m²)	33.7 ± 0.6	29.1 ± 2.1	**0.009**
Spacer period (days)	120.7 ± 13.3	91.8 ± 13.4	0.071

*BMI* body mass index, *SD* standard deviation, *P* probability value

### Changes in infection markers

Inflammatory parameters before and after spacer placement were evaluated.

At baseline (pre-spacer), CRP and ESR values were comparable between the handmade and prefabricated spacer groups, with no statistically significant differences observed (*p* = 0.431 and *p* = 0.083, respectively). However, WBC levels differed significantly between groups (*p* = 0.011).

In both groups, CRP, ESR, and WBC values showed significant reductions following spacer implantation (*p* < 0.01 for all comparisons).

When the magnitude of change (delta values) was compared between groups, no statistically significant differences were observed for CRP, ESR, or WBC (*p* = 0.488, *p* = 0.108, and *p* = 0.242, respectively). These findings suggest a comparable treatment response between the two groups (Table 2).

**Table 2:** .

Parameter	Handmade Spacer	*p*-value	Preformed Spacer	*p*-value
CRP (mg/L)	↓ Significant	0.013	NS	—
ESR (mm/h)	↓ Significant	0.035	NS	—
WBC (/µL)	↓ Significant	0.0006	NS	—

*CRP* C-reactive protein, *ESR* erythrocyte sedimentation rate, *WBC* white blood cell count, *NS* not significant, *P* probability value

#### Radiological Findings

Bone resorption values were higher in the handmade spacer group, although the differences were not statistically significant (femoral medial lysis: *p* = 0.188; tibial medial lysis: *p* = 0.244).

Medial joint space narrowing was greater in the handmade group (6.18 mm vs. 3.22 mm), approaching statistical significance (*p* = 0.098), while lateral gap narrowing showed no difference (*p* = 0.483).

The AORI scores were significantly higher in the handmade group, both for the femur (*p* = 0.005) and the tibia (*p* = 0.002), indicating more pronounced bone loss in these patients (Table 3).

**Table 3:** .

Parameter	Handmade Spacer	Preformed Spacer	*p*-value
Femoral medial lysis (mm)	Higher	Lower	0.188
Tibial medial lysis (mm)	Higher	Lower	0.244
Medial joint space narrowing (mm)	6.18 ± 2.4	3.22 ± 1.6	0.098
Femoral AORI score	Higher (F2–F3)	Lower (F1–F2)	**0.005**
Tibial AORI score	Higher (T2–T3)	Lower (T1–T2)	**0.002**

*AORI *Anderson Orthopaedic Research Institute, *mm * millimeter, *NS* not significant, *P* probability value

### Functional Outcomes

#### Functional evaluation suggested significantly superior outcomes in the prefabricated spacer group

Post-spacer range of motion (ROM) and WOMAC scores were significantly better in the prefabricated group (ROM: *p* = 0.007; WOMAC: *p* = 0.038).

Although post-reimplantation ROM did not reach statistical significance, the mean value was higher in the prefabricated group (79.4° vs. 65.0°; *p* = 0.080) (Table 4). No significant correlation was found between BMI and postoperative ROM (*r* = -0.149, *p* = 0.509) or WOMAC scores (*r* = -0.036, *p* = 0.870). When patients were categorized as BMI ≥ 30 and < 30, no significant differences were observed in ROM (*p* = 0.146) or WOMAC scores (*p* = 0.465).

**Table 4:** .

Parameter	Handmade Spacer	Preformed Spacer	*p*-value
ROM after spacer (°)	51.1 ± 4.8	61.7 ± 5.3	**0.007**
ROM after reimplantation (°)	65.0 ± 5.2	79.4 ± 6.1	0.080
WOMAC score	55.1 ± 5.7	41.2 ± 6.2	**0.038**

*ROM* range of motion, *WOMAC* Western Ontario and McMaster Universities Osteoarthritis Index, ° degrees, *NS* not significant, *P* probability value

### Ambulation status

Independent ambulation was achieved significantly more frequently in the prefabricated spacer group (77.8% vs. 21.4%; *p* = 0.019).

All immobilized patients were from the handmade spacer group (Table 5).

**Table 5:** .

Ambulation Level	Handmade Spacer	Preformed Spacer	*p*-value
Independent walking	21.4%	77.8%	**0.019**
Assisted walking	57.1%	22.2%	
Immobile	21.4%	0%	

*NS* not significant, *P* probability value

### Complications and clinical course

During the spacer period, two patients in the handmade group developed wound healing problems; one required flap reconstruction.

Long-term follow-up revealed two cases requiring salvage procedures due to failure of infection control and/or mechanical complications: one patient underwent Ilizarov arthrodesis and another required osteobridge reconstruction.

Apart from these, 21 patients completed treatment successfully without major complications.

### Correlation between AORI score and function

A significant negative correlation was found between AORI scores and functional parameters.

As AORI scores increased, ROM decreased, and WOMAC scores worsened (*p* < 0.05).

These findings suggest a potential association between increasing bone loss and worse postoperative functional outcomes.

## Discussion

This study compared the clinical, functional, and radiological outcomes of handmade and prefabricated spacers in patients undergoing two-stage revision surgery for periprosthetic knee joint infection. The findings suggest that the clinical pathway involving prefabricated articulating gentamicin-loaded spacers was associated with less bone lysis, lower reinfection rates, greater range of motion, and improved functional outcomes compared with handmade static vancomycin-loaded spacers. Additionally, prefabricated spacers were associated with fewer complications and superior overall clinical outcomes post-revision surgery. A unique aspect of this study was the direct radiological assessment of bone loss related to spacer use, systematically classified using the AORI scoring system. Consequently, the study objectively compared the long-term structural and functional impacts of spacer types, beyond merely infection control.

In this study, the clinical pathway involving prefabricated articulating gentamicin-loaded spacers was associated with more favorable clinical, radiological, and functional findings compared with handmade static vancomycin-loaded spacers. Although infection eradication was successful in both groups, the reinfection rate was 28.6% in the handmade group compared to 11.1% in the prefabricated group. Although the difference was not statistically significant, a numerical trend toward lower reinfection rates was observed in the prefabricated group. Literature similarly reports reinfection rates ranging from 0 to 2.7% for prefabricated spacers, with some series reporting no recurrence [11–13]. Several comparative studies have reported lower reinfection rates in prefabricated spacers (e.g., 1% versus 8%) [14], supporting a potential relationship between spacer type and infection control.

The possible advantage of prefabricated spacers in infection control could be attributed to their mechanical stability, homogeneous antibiotic release, and standardized surgical placement. Complex cases utilizing static spacers have been associated with higher infection recurrence rates, particularly in patients with high BMI or resistant microorganisms [15–17]. Recent comparative studies on revision strategies for chronic periprosthetic joint infection have similarly emphasized the importance of balancing infection eradication with preservation of function and postoperative recovery [18].Importantly, the differences observed between groups cannot be attributed solely to fabrication method, as spacer mobility (static vs. articulating) and antibiotic composition also differed between groups. Therefore, the results likely reflect a combined effect of spacer design, mobility, and antibiotic content rather than an isolated effect of “handmade versus prefabricated” spacer type. In addition, differences in antibiotic composition (vancomycin vs. gentamicin) may have influenced both infection control and the local bone environment through variations in antimicrobial spectrum and elution characteristics. Therefore, the observed differences between groups may partly reflect the combined effects of antibiotic properties in addition to spacer design and mobility.

In the present study, the handmade spacer group demonstrated a significantly higher body mass index, which may have contributed to increased mechanical stress, impaired wound healing, and inferior functional recovery. Due to the limited sample size, multivariate adjustment for BMI was not feasible; therefore, BMI should be considered a potential confounding factor when interpreting intergroup differences in radiological and functional outcomes. Future studies with larger cohorts and multivariable analyses are required to better clarify the independent effect of spacer type. Although a significant baseline difference in WBC levels was observed between groups, the magnitude of reduction in infection markers (CRP, ESR, and WBC) was similar. Therefore, differences in acute-phase response should be interpreted with caution.

Radiological assessments revealed more pronounced bone lysis in handmade spacer cases, especially notable in the femoral and tibial medial regions, reflected in higher AORI scores. Literature emphasizes the bone resorption risks associated with spacer types, suggesting that static spacers without intramedullary support may induce more significant bone loss due to embedding [19, 20]. Although prefabricated spacers generally show better bone preservation, some studies have reported non-significant differences [21]. Our study methodologically contributes to the literature through standardized radiological measurements and systematic use of the AORI classification.

Functional evaluations indicated significantly better ROM and WOMAC scores in the prefabricated spacer group compared to handmade spacers. These findings align with numerous studies reporting superior ROM and patient satisfaction with articulating spacers [22–24]. Literature shows average ROM values of 111° for functional spacers versus 82° for static spacers [25], closely paralleling our study’s findings (79.4° prefabricated vs. 65° handmade). Mobility assessments further supported these functional benefits, as prefabricated spacers facilitated earlier independent ambulation. This functional advantage may be primarily related to the articulating nature of the prefabricated spacer rather than the fabrication method itself. Functional outcomes such as ROM, WOMAC scores, and ambulation status are likely multifactorial and may also be influenced by residual bone loss, postoperative pain, soft tissue balance, rehabilitation capacity, and patient-related factors in addition to spacer characteristics. Recent literature has similarly emphasized that postoperative pain distribution and functional recovery following knee arthroplasty are influenced by multiple biomechanical and patient-related factors beyond implant configuration alone [26]. 

Handmade spacers exhibited notably greater medial gap narrowing, suggesting potential complications such as soft tissue contracture and impaired joint mechanics. Literature indicates that articulating and prefabricated spacers offer better joint gap preservation, simplifying component placement in subsequent surgeries and enhancing functional outcomes [23, 27]. However, early postoperative radiographs in our study may have partially underestimated gap measurements due to incomplete weight-bearing from patient pain. Despite this limitation, average gap differences remain clinically relevant.

Complication analysis revealed more adverse outcomes in the handmade spacer group, including arthrodesis, Ilizarov fixation, spacer insufficiency, and wire breakage. Literature also associates static and molded spacers with higher complication rates, sometimes exceeding 30%, compared to 10–15% for prefabricated spacers [28, 29]. Consistent with literature, our study found fewer complications and less frequent need for non-revision interventions in the prefabricated group.

The AORI classification not only assesses bone loss but directly impacts surgical complexity and implant selection [30, 31]. Higher AORI types (F3, T3) typically require extended surgical duration and more constrained prostheses or augments. The higher frequency of F3/T3 scores in our handmade spacer group indicates increased complexity in subsequent surgeries, emphasizing that spacer selection critically influences both infection control and future surgical planning. Although the AORI classification is primarily used intraoperatively, it was applied in this study as a practical framework to describe the radiographic severity of bone defects. In this study, AORI classification was used at two different time points to assess the progression of bone loss during the spacer period, providing a clinically relevant framework for evaluating structural changes between stages.

Strengths of this study include systematic evaluation and comparison of infection control, radiological bone loss, and functional outcomes. Bone lysis, often overlooked or subjectively assessed, was objectively measured using standardized radiographic references. Additionally, structural bone loss was classified using the AORI system, clarifying its clinical implications for surgical planning. Comprehensive comparison of functional outcomes (ROM, WOMAC, ambulation) and detailed complication analysis further enhances clinical guidance. Importantly, all patients underwent reimplantation using the same prosthesis design and constraint level, which minimized the potential influence of implant-related factors on postoperative ROM and WOMAC outcomes.

A major limitation of this study is the lack of randomization in spacer selection. Although spacer type was not intentionally assigned based on disease severity or case complexity, selection was influenced by surgeon preference and implant availability. Therefore, confounding by indication cannot be excluded. It is possible that unrecognized baseline differences, such as bone loss severity or soft tissue condition, may have influenced the observed outcomes. Consequently, the results should be interpreted with caution, and the findings should be considered hypothesis-generating rather than definitive. Radiographic measurements were performed by a single observer without assessment of intraobserver or interobserver reliability, which may affect the robustness of the findings. Although patients with sinus tract and major soft tissue defects were excluded, other factors such as organism virulence and antibiotic resistance profiles were not standardized. This may have contributed to residual confounding related to baseline infection severity.In addition, the relatively small sample size limits the statistical power of the study and may affect the stability of both positive and negative findings. Therefore, some of the observed differences may represent numerical trends rather than definitive conclusions. These findings should be interpreted with caution and considered hypothesis-generating. Additional analyses did not demonstrate a significant association between BMI and functional outcomes, suggesting that BMI alone is unlikely to be a major confounder influencing intergroup differences. However, given the limited sample size, these findings should be interpreted with caution.

Future prospective or randomized studies are required to better isolate the independent effect of spacer type.

## Conclusion

In this retrospective cohort, the clinical pathway involving prefabricated articulating gentamicin-loaded spacers was associated with less radiological bone loss and improved functional outcomes compared with handmade static vancomycin-loaded spacers. However, these findings may reflect the combined influence of spacer design, mobility, and antibiotic composition rather than fabrication method alone, and should therefore be interpreted as hypothesis-generating.

## Data Availability

The datasets generated and analyzed during the current study are available from the corresponding author on reasonable request.
